# An autophagy‐related long non‐coding RNA signature for glioma

**DOI:** 10.1002/2211-5463.12601

**Published:** 2019-03-05

**Authors:** Fangkun Luan, Wenjie Chen, Miao Chen, Jun Yan, Hao Chen, Haiyue Yu, Tieqi Liu, Ligen Mo

**Affiliations:** ^1^ Department of Neurosurgery Affiliated Tumor Hospital of Guangxi Medical University Nanning China; ^2^ Department of Ultrasound Affiliated Tumor Hospital of Guangxi Medical University Nanning China

**Keywords:** autophagy, CCGA, glioma, long non‐coding RNA, prognostic signature, TCGA

## Abstract

Glioma is one of the most common types of malignant primary central nervous system tumor, and prognosis for this disease is poor. As autophagic drugs have been reported to induce glioma cell death, we investigated the potential prognostic role of autophagy‐associated long non‐coding RNA (lncRNA) in glioma patients. In this study, we obtained 879 lncRNAs and 216 autophagy genes from the Chinese Glioma Genome Atlas microarray, and found that 402 lncRNAs are correlated with the autophagy genes. Subsequently, 10 autophagy‐associated lncRNAs with prognostic value (*PCBP1‐AS1*,* TP53TG1*,* DHRS4‐AS1*,* ZNF674‐AS1*,* GABPB1‐AS1*,* DDX11‐AS1*,* SBF2‐AS1*,* MIR4453HG*,* MAPKAPK5*‐AS1 and *COX10‐AS1*) were identified in glioma patients using multivariate Cox regression analyses. A prognostic signature was then established based on these prognostic lncRNAs, dividing patients into low‐risk and high‐risk groups. The overall survival time was shorter in the high‐risk group than that in the low‐risk group [hazard ratio (HR) = 5.307, 95% CI: 4.195–8.305; *P* < 0.0001]. Gene set enrichment analysis revealed that the gene sets were significantly enriched in cancer‐related pathways, including interleukin (IL) 6/Janus kinase/signal transducer and activator of transcription (STAT) 3 signaling, tumor necrosis factor α signaling via nuclear factor κB, IL2/STAT5 signaling, the p53 pathway and the KRAS signaling pathway. The Cancer Genome Atlas dataset was used to validate that high‐risk patients have worse survival outcomes than low‐risk patients (HR = 1.544, 95% CI: 1.110–2.231; *P* = 0.031). In summary, our signature of 10 autophagy‐related lncRNAs has prognostic potential for glioma, and these autophagy‐related lncRNAs may play a key role in glioma biology.

**Abbreviations**
CGGA: Chinese Glioma Genome Atlas
GBM: glioblastoma
GSEA: gene set enrichment analysis
HCC: hepatocellular carcinoma
*HOTAIR*: *HOX* (homeobox) transcript antisense RNA
HR: hazard ratio
IFN: interferon
IL: interleukin
lncRNA: long non‐coding RNA
miRNA: microRNA
MMP: matrix metalloproteinase
OS: overall survival
STAT: signal transducer and activator of transcription
TCGA: The Cancer Genome Atlas

Glioma is one of the most common types of malignant primary central nervous system tumors with poor prognosis, comprising approximately 44% of central nervous system tumors 1. The prognosis of glioblastoma (GBM) is the worst among gliomas, in which the median overall survival (OS) for patients with GBM is 15–23 months and the 5‐year survival rate is less than 6% 2, 3. Upon diagnosis, the standard treatment of glioma includes maximal surgical resection, chemotherapy, such as temozolomide, and radiation. Treatment options may vary in different stages of the disease and by the age of the patients. Various factors affect the prognosis of GBM including *EGFR* amplifications, and mutations of *IDH1*,* TP53* and *PTEN* 4, 5, 6. However, the survival outcome is unfavorable due to the complex genetic mechanism.

Autophagy is the physiological process that directs degradation of proteins and whole organelles in cells. The activation of autophagy is divided into normal and pathological conditions. Under normal circumstances, autophagy represents a response to several stresses by providing the necessary circulating metabolic substrates for survival. In addition, autophagy is active in some pathological processes in order to maintain cellular homeostasis, such as neurodegenerative diseases, pathogenic inflammation, aging and cancer 7. In recent years, many studies have sought to find new potential targeted therapies by investigating autophagy pathways 8, 9, 10. In addition, autophagic drugs induce cell autophagic death (type II cell death) and cause glioma cell death. Whether this is an alternative and emerging concept for the study of novel glioma therapies remains largely unknown 11.

Long non‐coding RNAs (lncRNAs) have a wide range of functional activities 12. They play a significant role in physiological processes, including RNA decay, genetic regulation of gene expression, RNA splicing, microRNA (miRNA) regulation and protein folding 13. lncRNA regulates many proteins that are important for autophagy. Impaired functioning of lncRNAs participates in glioma pathogenesis, such as cellular apoptosis and proliferation 14. *HOX* (homeobox) transcript antisense RNA (*HOTAIR*) is a lncRNA that plays an important role in the regulation of cancer transformation, mainly due to extensive miRNA–*HOTAIR* interactions and its effect on matrix metalloproteinases (MMPs) 15. There may be an involvement of *HOTAIR*‐interacting miRNAs and MMPs in autophagy regulation 16, 17, 18. Sufficient evidence shows that lncRNAs mediate transcriptional and post‐transcriptional levels of autophagy‐related genes to regulate the autophagy regulatory network 19, 20. This paper proposes to construct a coexpression network of autophagy‐related lncRNAs using bioinformatics methods, providing a theoretical basis for the treatment of gliomas 21.

Therefore, autophagy‐related lncRNAs may have potential value in the prognosis of glioma patients and may serve as potential therapeutic targets. Here, we aimed to establish an autophagy‐related lncRNA signature in glioma and to advance the targeted treatment of glioma.

## Materials and methods

### Information extraction of glioma patients

The Chinese Glioma Genome Atlas (CGGA, http://www.cgga.org.cn/, freely available) microarray was used as a training set to establish an autophagy‐associated lncRNA signature of glioma patients. CGGA is the largest glioma tissue database with follow‐ups in China. Thousands of samples have been subjected to whole‐exome sequencing, DNA methylation microarray detection and whole‐genome sequencing, miRNA, mRNA and circRNA sequencing. The training dataset includes CGGA mRNA expression (FPKM) in 325 glioma patients together with relevant clinical data. The patients were diagnosed based on the 2007 WHO classification guidelines. We downloaded clinical information from the dataset website. The prognostic signature was further validated based on The Cancer Genome Atlas (TCGA, https://cancergenome.nih.gov/) GBM dataset. TCGA GBM dataset (FPKM level 3) was included in our analysis as a validation dataset with 160 GBM patients.

### LncRNA and autophagy gene screening

The profiles of lncRNAs and autophagy genes were obtained from the CGGA ALL mRNAseq dataset. Specifically, the autophagy gene list was obtained from the Human Autophagy Database (HADb, http://autophagy.lu/clustering/index.html). All of the mRNA expression data were normalized by log2 transformation. Pearson correlation was applied to calculate the correlation between the lncRNAs and autophagy‐related genes. A lncRNA with a correlation coefficient |*R* ^2^| > 0.3 and *P* < 0.05 was considered to be an autophagy‐related lncRNA.

### Signature development

First, univariate and multivariate Cox regression analyses were performed to evaluate the prognostic value of autophagy‐related lncRNAs. The lncRNAs with a *P*‐value < 0.01 by univariate analysis were included in the multivariate stepwise regression Cox analysis to establish the risk score. We used the previous report to determine the risk score for each patient using the following formula: Risk score = β_gene1_ × expr_gene1_ + β_gene2_ × expr_gene2_ + ··· +  β_gene*n*_ × expr_gene*n*_. Cox analysis was performed to build a signature for predicting survival. For more detail, we assigned risk scores by a linear combination of the expression levels of lncRNAs weighted by regression coefficients (β). The β value was calculated by log transformation of the hazard ratio (HR) from the multivariate Cox regression analysis. High‐risk and low‐risk groups were established based on the median risk score. The lncRNA expression is defined as expr_gene*n*_.

### Gene set enrichment analysis

Gene set enrichment analysis (GSEA) was used to interpret gene expression data. This method derives its function by analyzing gene sets, so it can be used to determine whether the gene set shows a statistically significant difference between the two biological states. In this study, we verified whether genes that are differentially expressed between two groups are enriched during autophagy.

### Statistical analysis

The expression levels of autophagy‐related lncRNAs were elevated (*P* ≤ 0.05). Construction of the autophagy–lncRNA coexpression network was completed using cytoscape software 22 (version 3.4.0; The Cytoscape Consortium, San Diego, CA, USA). Pearson correlation analysis and Cox regression analysis were performed using spss statistics software (version 24; IBM Corp., Armonk, NY, USA). Survival status was the basis for univariate cox regression analysis. prism 7 (GraphPad Software Inc., La Jolla, CA, USA) was used to generate Kaplan–Meier curves. GSEA ( http://www.broadinstitute.org/gsea/index.jsp) was used to distinguish between two sets of functional annotations. Statistical significance was set at a threshold of a two‐tailed *P* < 0.05.

## Results

### Construction of a coexpression network for autophagy–lncRNAs

We identified a total of 878 lncRNAs in the CGGA dataset, which was extracted from the CGGA database. A total of 215 autophagy‐related genes were extracted from the Human Autophagy Database (HADb, http://autophagy.lu/clustering/index.html). We constructed an autophagy–lncRNA coexpression network to identify autophagy‐related lncRNAs. Finally, 402 lncRNAs were identified (|*R* ^2^| > 0.3 and *P* ≤ 0.05).

### Identification of a signature of 10 autophagy‐related lncRNAs in patients with glioma

**Figure 1:**
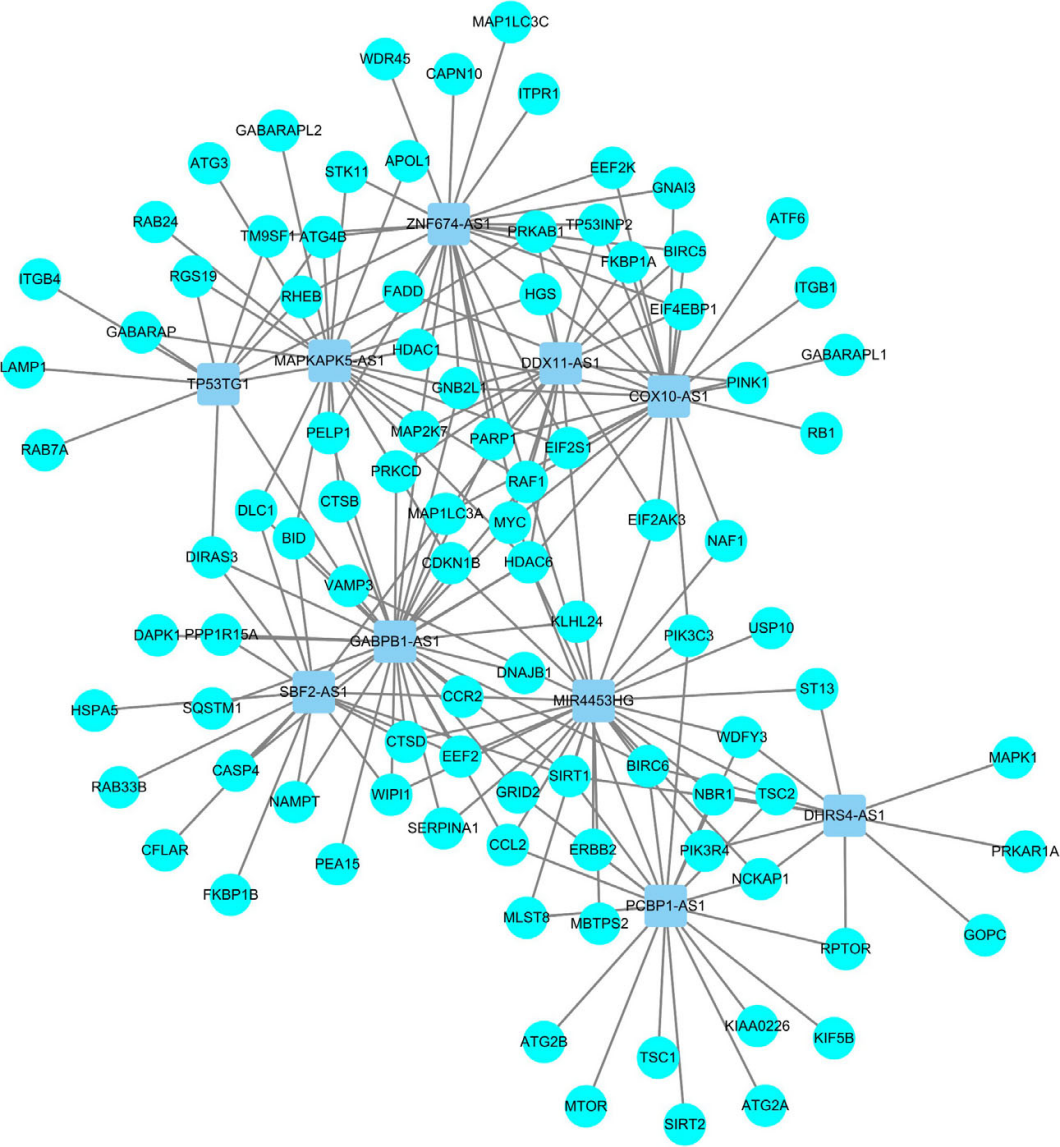
Network of prognostic lncRNAs with co‐expressed autophagy genes in glioma. In the centric position, grey blue nodes indicate lncRNAs and the sky blue indicates autophagy genes. The coexpression network is visualized by cytoscape 3.4 software.

**Figure 2:**
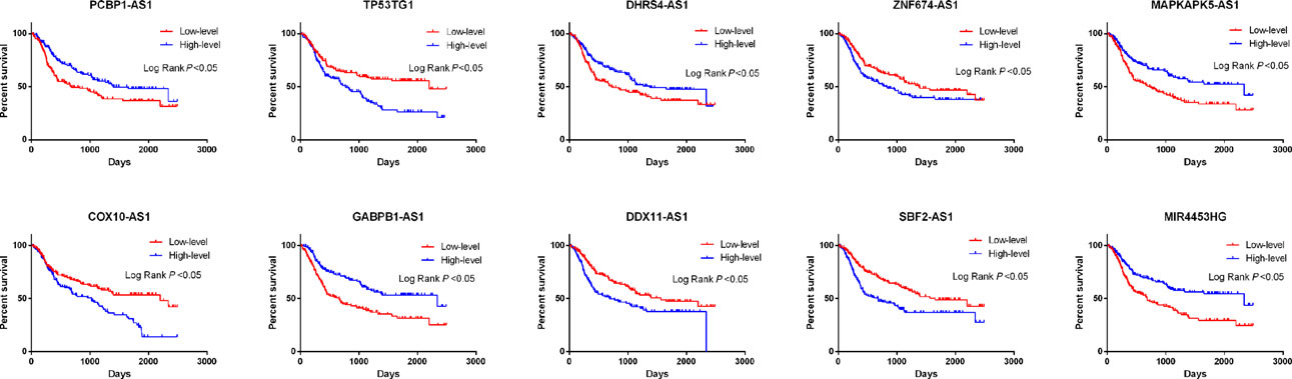
Kaplan–Meier survival curves for the 10 prognostic lncRNAs for glioma in CCGA dataset. The 10 autophagy‐related lncRNAs were found to be independent prognostic factors for glioma patients, of which five lncRNAs were unfavorable factors (*TP53TG1*,* ZNF674‐AS1*,* COX10‐AS1*,* DDX11‐AS1* and *SBF2‐AS1*) and five lncRNAs were confirmed to be favorable prognostic factors for glioma (*PCBP1‐AS1*,* DHRS4‐AS1*,* GABPB1‐AS1*,* MAPKAPK5‐AS1* and *MIR4453HG*).

First, we identified autophagy‐related lncRNAs by constructing autophagy–lncRNA coexpression networks (*P* ≤ 0.05). In addition, we used univariate Cox regression analysis based on 402 autophagy‐associated lncRNAs to screen prognostic genes. We ranked the prognostic autophagy‐related lncRNAs in ascending order by their *P* values. We used a *P* value of 0.05 as the cutoff value, and the lncRNAs that satisfied this were used for signature development. Our training set was a collection of 325 glioma patients from the CGGA dataset. A total of 19 lncRNAs have prognostic value for glioma patients (*P* < 0.01). Subsequently, 10 autophagy‐related lncRNAs were found to be independent prognostic factors for glioma patients (Table 1 and Fig. 1), of which five lncRNAs were unfavorable factors (*TP53TG1*,* ZNF674‐AS1*,* COX10‐AS1*,* DDX11‐AS1* and *SBF2‐AS1*) and five lncRNAs were confirmed to be favorable prognostic factors for glioma (*PCBP1‐AS1*,* DHRS4‐AS1*,* GABPB1‐AS1*,* MAPKAPK5‐AS1* and *MIR4453HG*) (Table 2 and Fig. 2).

**Table 1:** Correlation between the prognostic lncRNAs and autophagy genes in glioma

LncRNA	Autophagy gene	Correlation	*P*
*PCBP1‐AS1*	*CCL2*	−0.360049202	2.2072E‐11
*PCBP1‐AS1*	*ATG2B*	0.304817007	2.0467E‐08
*PCBP1‐AS1*	*KIF5B*	0.305876376	1.818E‐08
*PCBP1‐AS1*	*ATG2A*	0.311692494	9.4056E‐09
*PCBP1‐AS1*	*WDFY3*	0.31817447	4.4365E‐09
*PCBP1‐AS1*	*MLST8*	0.322140176	2.7765E‐09
*PCBP1‐AS1*	*PIK3C3*	0.324642153	2.0586E‐09
*PCBP1‐AS1*	*TSC1*	0.330291284	3.8213E‐09
*PCBP1‐AS1*	*TSC2*	0.332514623	8.3751E‐10
*PCBP1‐AS1*	*NCKAP1*	0.339888292	3.3348E‐10
*PCBP1‐AS1*	*GRID2*	0.364527555	5.5069E‐11
*PCBP1‐AS1*	*SIRT2*	0.366890173	8.5922E‐12
*PCBP1‐AS1*	*MTOR*	0.371080649	4.7675E‐12
*PCBP1‐AS1*	*SIRT1*	0.385311738	6.0485E‐13
*PCBP1‐AS1*	*NBR1*	0.3900753	3.5039E‐13
*PCBP1‐AS1*	*ERBB2*	0.398520286	8.1268E‐14
*PCBP1‐AS1*	*BIRC6*	0.401737956	4.9072E‐14
*PCBP1‐AS1*	*HDAC6*	0.410137987	1.2879E‐14
*PCBP1‐AS1*	*KIAA0226*	0.417146773	3.9968E‐15
*PCBP1‐AS1*	*RPTOR*	0.426641207	8.8818E‐16
*TP53TG1*	*GABARAP*	0.44046552	0
*TP53TG1*	*TM9SF1*	0.440120961	0
*TP53TG1*	*VAMP3*	0.43087095	4.4409E‐16
*TP53TG1*	*ITGB4*	0.379361437	1.4515E‐12
*TP53TG1*	*LAMP1*	0.362507507	1.5766E‐11
*TP53TG1*	*RHEB*	0.338549143	3.7103E‐10
*TP53TG1*	*FADD*	0.322540605	2.6472E‐09
*TP53TG1*	*RAB7A*	0.320497198	3.3743E‐09
*TP53TG1*	*HDAC1*	0.316709215	5.2662E‐09
*TP53TG1*	*ATG4B*	0.310888419	1.0312E‐08
*TP53TG1*	*DIRAS3*	0.305503826	1.8954E‐08
*TP53TG1*	*RGS19*	0.300279741	3.5506E‐08
*DHRS4‐AS1*	*PRKAR1A*	0.304280356	2.6545E‐08
*DHRS4‐AS1*	*BIRC6*	0.307830991	1.7911E‐08
*DHRS4‐AS1*	*RPTOR*	0.313316207	9.6535E‐09
*DHRS4‐AS1*	*WDFY3*	0.314945434	8.0148E‐09
*DHRS4‐AS1*	*GOPC*	0.319627801	5.2128E‐09
*DHRS4‐AS1*	*MAPK1*	0.320002607	4.4667E‐09
*DHRS4‐AS1*	*ST13*	0.322382713	3.3795E‐09
*DHRS4‐AS1*	*PIK3R4*	0.323543353	2.9471E‐09
*DHRS4‐AS1*	*NCKAP1*	0.367547056	1.0542E‐11
*DHRS4‐AS1*	*SIRT1*	0.40099047	7.8826E‐14
*DHRS4‐AS1*	*NBR1*	0.430691637	6.6613E‐16
*ZNF674‐AS1*	*ITPR1*	−0.319258764	1.3176E‐08
*ZNF674‐AS1*	*TP53INP2*	−0.313961307	7.2452E‐09
*ZNF674‐AS1*	*EIF2S1*	0.304321171	2.163E‐08
*ZNF674‐AS1*	*PARP1*	0.311082339	1.0086E‐08
*ZNF674‐AS1*	*HDAC1*	0.311370172	9.7591E‐09
*ZNF674‐AS1*	*EEF2K*	0.319838206	3.6476E‐09
*ZNF674‐AS1*	*TM9SF1*	0.321374147	3.0412E‐09
*ZNF674‐AS1*	*GNAI3*	0.331509862	8.9278E‐10
*ZNF674‐AS1*	*FKBP1A*	0.339350907	3.3524E‐10
*ZNF674‐AS1*	*ATG4B*	0.34228221	2.308E‐10
*ZNF674‐AS1*	*PELP1*	0.342564645	2.226E‐10
*ZNF674‐AS1*	*FADD*	0.344531987	1.7285E‐10
*ZNF674‐AS1*	*ENSG00000177993.3*	0.344786314	1.6727E‐10
*ZNF674‐AS1*	*HGS*	0.349662833	8.8614E‐11
*ZNF674‐AS1*	*WDR45*	0.350583974	7.8496E‐11
*ZNF674‐AS1*	*CAPN10*	0.357666059	3.0499E‐11
*ZNF674‐AS1*	*RHEB*	0.357722928	3.0266E‐11
*ZNF674‐AS1*	*MAP1LC3C*	0.358011106	2.9109E‐11
*ZNF674‐AS1*	*BIRC5*	0.365800568	3.2339E‐11
*ZNF674‐AS1*	*MAP2K7*	0.37311006	3.5731E‐12
*ZNF674‐AS1*	*STK11*	0.375183168	2.6561E‐12
*ZNF674‐AS1*	*GNB2L1*	0.402865027	4.1078E‐14
*ZNF674‐AS1*	*PRKAB1*	0.411848633	9.77E‐15
*ZNF674‐AS1*	*EIF4EBP1*	0.441387008	0
*ZNF674‐AS1*	*RAF1*	0.471527103	0
*ZNF674‐AS1*	*HDAC6*	0.529212582	0
*MAPKAPK5‐AS1*	*DLC1*	−0.37599408	2.7613E‐12
*MAPKAPK5‐AS1*	*CTSB*	−0.321021809	3.1711E‐09
*MAPKAPK5‐AS1*	*RGS19*	−0.304801176	2.1559E‐08
*MAPKAPK5‐AS1*	*PRKCD*	−0.303327412	7.978E‐08
*MAPKAPK5‐AS1*	*APOL1*	−0.301552957	5.851E‐08
*MAPKAPK5‐AS1*	*EIF2S1*	0.306873043	1.6255E‐08
*MAPKAPK5‐AS1*	*STK11*	0.316360528	5.4847E‐09
*MAPKAPK5‐AS1*	*ATG3*	0.317029326	5.073E‐09
*MAPKAPK5‐AS1*	*GNB2L1*	0.325707112	1.8109E‐09
*MAPKAPK5‐AS1*	*PRKAB1*	0.332894214	7.5251E‐10
*MAPKAPK5‐AS1*	*MAP2K7*	0.334240467	6.3674E‐10
*MAPKAPK5‐AS1*	*ATG4B*	0.342782716	2.1647E‐10
*MAPKAPK5‐AS1*	*GABARAPL2*	0.344580777	1.7176E‐10
*MAPKAPK5‐AS1*	*RAF1*	0.368047755	7.3084E‐12
*MAPKAPK5‐AS1*	*HDAC6*	0.370823221	4.9445E‐12
*MAPKAPK5‐AS1*	*HGS*	0.373857161	3.2117E‐12
*MAPKAPK5‐AS1*	*BID*	0.396105786	1.1813E‐13
*MAPKAPK5‐AS1*	*RAB24*	0.404333356	3.2641E‐14
*MAPKAPK5‐AS1*	*GABARAP*	0.420904922	2.2204E‐15
*MAPKAPK5‐AS1*	*PELP1*	0.451556383	0
*MAPKAPK5‐AS1*	*CDKN1B*	0.456366201	0
*COX10‐AS1*	*MAP1LC3A*	−0.375521773	2.53E‐12
*COX10‐AS1*	*TP53INP2*	−0.369627107	5.854E‐12
*COX10‐AS1*	*PINK1*	−0.367447805	7.9483E‐12
*COX10‐AS1*	*GABARAPL1*	−0.345745019	1.4775E‐10
*COX10‐AS1*	*HDAC1*	0.30053166	3.2895E‐08
*COX10‐AS1*	*FKBP1A*	0.305650956	1.8644E‐08
*COX10‐AS1*	*RB1*	0.307329731	1.544E‐08
*COX10‐AS1*	*ATF6*	0.308265668	1.3892E‐08
*COX10‐AS1*	*HGS*	0.312518629	8.5551E‐09
*COX10‐AS1*	*NAF1*	0.313398471	7.7313E‐09
*COX10‐AS1*	*PIK3C3*	0.323065939	2.4864E‐09
*COX10‐AS1*	*ITGB1*	0.330112011	1.0601E‐09
*COX10‐AS1*	*PRKAB1*	0.333014795	7.4136E‐10
*COX10‐AS1*	*GNB2L1*	0.344926977	1.6425E‐10
*COX10‐AS1*	*PARP1*	0.345318807	1.5614E‐10
*COX10‐AS1*	*EIF2S1*	0.3460972	1.4116E‐10
*COX10‐AS1*	*FADD*	0.361593998	1.7871E‐11
*COX10‐AS1*	*MYC*	0.375188431	2.6541E‐12
*COX10‐AS1*	*EEF2K*	0.377661311	1.8581E‐12
*COX10‐AS1*	*EIF4EBP1*	0.405855635	2.5757E‐14
*COX10‐AS1*	*EIF2AK3*	0.419195952	2.8866E‐15
*COX10‐AS1*	*HDAC6*	0.428787718	4.4409E‐16
*COX10‐AS1*	*GNAI3*	0.439816001	0
*COX10‐AS1*	*BIRC5*	0.448936102	0
*COX10‐AS1*	*RAF1*	0.572860513	0
*GABPB1‐AS1*	*BID*	0.420118584	2.4425E‐15
*GABPB1‐AS1*	*BIRC6*	0.36038314	2.1089E‐11
*GABPB1‐AS1*	*CASP4*	−0.343935388	5.2362E‐10
*GABPB1‐AS1*	*CCL2*	−0.373849011	3.2156E‐12
*GABPB1‐AS1*	*CCR2*	−0.368177579	3.6858E‐11
*GABPB1‐AS1*	*CDKN1B*	0.364553409	1.1889E‐11
*GABPB1‐AS1*	*CTSB*	−0.372819935	3.7241E‐12
*GABPB1‐AS1*	*CTSD*	−0.43970168	0
*GABPB1‐AS1*	*DAPK1*	0.323366578	2.3986E‐09
*GABPB1‐AS1*	*DIRAS3*	−0.300618621	3.2582E‐08
*GABPB1‐AS1*	*DLC1*	−0.313694777	8.3115E‐09
*GABPB1‐AS1*	*DNAJB1*	−0.308644775	1.3309E‐08
*GABPB1‐AS1*	*EEF2*	0.36428838	1.2333E‐11
*GABPB1‐AS1*	*GNB2L1*	0.304868539	2.0349E‐08
*GABPB1‐AS1*	*GRID2*	0.358164661	1.2447E‐10
*GABPB1‐AS1*	*HDAC6*	0.583737387	0
*GABPB1‐AS1*	*KLHL24*	0.37211066	4.1194E‐12
*GABPB1‐AS1*	*MAP1LC3A*	−0.335686931	5.3165E‐10
*GABPB1‐AS1*	*MAP2K7*	0.351129758	7.3042E‐11
*GABPB1‐AS1*	*MYC*	0.350474546	7.9636E‐11
*GABPB1‐AS1*	*NAMPT*	−0.317006934	5.0863E‐09
*GABPB1‐AS1*	*PARP1*	0.318853967	4.0962E‐09
*GABPB1‐AS1*	*PEA15*	0.306793364	1.6401E‐08
*GABPB1‐AS1*	*PELP1*	0.350174812	8.2843E‐11
*GABPB1‐AS1*	*PPP1R15A*	−0.432497553	4.4409E‐16
*GABPB1‐AS1*	*PRKCD*	−0.354522637	2.4201E‐10
*GABPB1‐AS1*	*RAF1*	0.518711717	0
*GABPB1‐AS1*	*SERPINA1*	−0.35246404	9.1958E‐11
*GABPB1‐AS1*	*SIRT1*	0.377399334	1.9298E‐12
*GABPB1‐AS1*	*SQSTM1*	−0.300514253	3.2958E‐08
*GABPB1‐AS1*	*VAMP3*	−0.349193236	9.4249E‐11
*GABPB1‐AS1*	*WIPI1*	−0.425794454	8.8818E‐16
*DDX11‐AS1*	*PINK1*	−0.380737586	1.1873E‐12
*DDX11‐AS1*	*PRKCD*	−0.337496704	1.8761E‐09
*DDX11‐AS1*	*MAP1LC3A*	−0.321966423	2.8346E‐09
*DDX11‐AS1*	*TP53INP2*	−0.315547251	6.0291E‐09
*DDX11‐AS1*	*EIF2AK3*	0.301487968	2.9609E‐08
*DDX11‐AS1*	*EIF2S1*	0.34484361	1.6603E‐10
*DDX11‐AS1*	*FKBP1A*	0.36028176	2.1383E‐11
*DDX11‐AS1*	*MAP2K7*	0.361662635	1.7704E‐11
*DDX11‐AS1*	*MYC*	0.36701736	8.4412E‐12
*DDX11‐AS1*	*PRKAB1*	0.367277985	8.1393E‐12
*DDX11‐AS1*	*HGS*	0.398721365	7.8604E‐14
*DDX11‐AS1*	*PARP1*	0.399014394	7.5051E‐14
*DDX11‐AS1*	*GNB2L1*	0.446121011	0
*DDX11‐AS1*	*HDAC6*	0.4630659	0
*DDX11‐AS1*	*BIRC5*	0.494242839	0
*DDX11‐AS1*	*EIF4EBP1*	0.494327951	0
*DDX11‐AS1*	*RAF1*	0.595959165	0
*SBF2‐AS1*	*SIRT1*	−0.359724478	2.6578E‐11
*SBF2‐AS1*	*BID*	−0.359318057	2.8079E‐11
*SBF2‐AS1*	*EEF2*	−0.345315983	1.7787E‐10
*SBF2‐AS1*	*HDAC6*	−0.30180643	3.1545E‐08
*SBF2‐AS1*	*HSPA5*	0.303106799	2.7341E‐08
*SBF2‐AS1*	*NAMPT*	0.305876474	2.0115E‐08
*SBF2‐AS1*	*DLC1*	0.313900704	9.0314E‐09
*SBF2‐AS1*	*PPP1R15A*	0.315700878	6.5975E‐09
*SBF2‐AS1*	*FKBP1B*	0.330524677	1.1347E‐09
*SBF2‐AS1*	*WIPI1*	0.347602864	1.3239E‐10
*SBF2‐AS1*	*DIRAS3*	0.34895202	1.111E‐10
*SBF2‐AS1*	*CFLAR*	0.353930283	5.7749E‐11
*SBF2‐AS1*	*CCR2*	0.365426608	6.1051E‐11
*SBF2‐AS1*	*CASP4*	0.387535164	1.7764E‐12
*SBF2‐AS1*	*CTSD*	0.39811287	1.0303E‐13
*SBF2‐AS1*	*RAB33B*	0.41262521	1.0436E‐14
*SBF2‐AS1*	*MAP1LC3A*	0.466078431	0
*MIR4453HG*	*BIRC6*	0.482298774	0
*MIR4453HG*	*CCL2*	−0.32967133	1.1188E‐09
*MIR4453HG*	*CCR2*	−0.306263916	5.3174E‐08
*MIR4453HG*	*CDKN1B*	0.37854434	1.6347E‐12
*MIR4453HG*	*CTSD*	−0.36405224	1.2743E‐11
*MIR4453HG*	*EEF2*	0.339277778	3.3836E‐10
*MIR4453HG*	*EIF2AK3*	0.30731381	1.5468E‐08
*MIR4453HG*	*EIF2S1*	0.300378118	3.3454E‐08
*MIR4453HG*	*ERBB2*	0.31509245	6.356E‐09
*MIR4453HG*	*GRID2*	0.33900455	1.3033E‐09
*MIR4453HG*	*HDAC6*	0.530273615	0
*MIR4453HG*	*MBTPS2*	0.372080137	4.1376E‐12
*MIR4453HG*	*MLST8*	0.336179553	4.9987E‐10
*MIR4453HG*	*NAF1*	0.343120261	2.0729E‐10
*MIR4453HG*	*NBR1*	0.332149604	9.3012E‐10
*MIR4453HG*	*NCKAP1*	0.315032585	6.7536E‐09
*MIR4453HG*	*PIK3C3*	0.398066735	8.7041E‐14
*MIR4453HG*	*PIK3R4*	0.383400705	8.0247E‐13
*MIR4453HG*	*RAF1*	0.446543819	0
*MIR4453HG*	*SERPINA1*	−0.316157599	7.7713E‐09
*MIR4453HG*	*SIRT1*	0.394985589	1.4033E‐13
*MIR4453HG*	*ST13*	0.343209924	2.0492E‐10
*MIR4453HG*	*TSC2*	0.324952539	2.1001E‐09
*MIR4453HG*	*USP10*	0.330643906	1.3364E‐09
*MIR4453HG*	*VAMP3*	−0.34420749	1.8024E‐10
*MIR4453HG*	*WDFY3*	0.321627435	2.9511E‐09
*MIR4453HG*	*WIPI1*	−0.324415393	2.1154E‐09

**Table 2:** Detailed information for 10 autophagy‐related lncRNAs significantly associated with OS in glioma

LncRNA	Ensemble ID	β	SE	*P*	HR	Lower	Upper
*PCBP1‐AS1*	ENSG00000179818	−0.363	0.184	0.049	0.696	0.485	0.998
*TP53TG1*	ENSG00000182165	0.443	0.16	0.006	1.558	1.139	2.131
*DHRS4‐AS1*	ENSG00000215256	−0.253	0.099	0.01	0.776	0.639	0.942
*ZNF674‐AS1*	ENSG00000230844	0.448	0.199	0.024	1.565	1.06	2.31
*MAPKAPK5‐AS1 *	ENSG00000234608	−0.64	0.245	0.009	0.527	0.326	0.852
*COX10‐AS1*	ENSG00000236088	0.829	0.194	< 0.001	2.29	1.565	3.351
*GABPB1‐AS1*	ENSG00000244879	−0.403	0.154	0.009	0.668	0.494	0.904
*DDX11‐AS1 *	ENSG00000245614	0.296	0.128	0.021	1.344	1.046	1.726
*SBF2‐AS1 *	ENSG00000246273	0.134	0.064	0.036	1.143	1.009	1.295
*MIR4453HG*	ENSG00000268471	−0.551	0.155	< 0.001	0.577	0.426	0.781

### The prognostic impact of an autophagy‐related lncRNA signature for glioma

**Figure 3:**
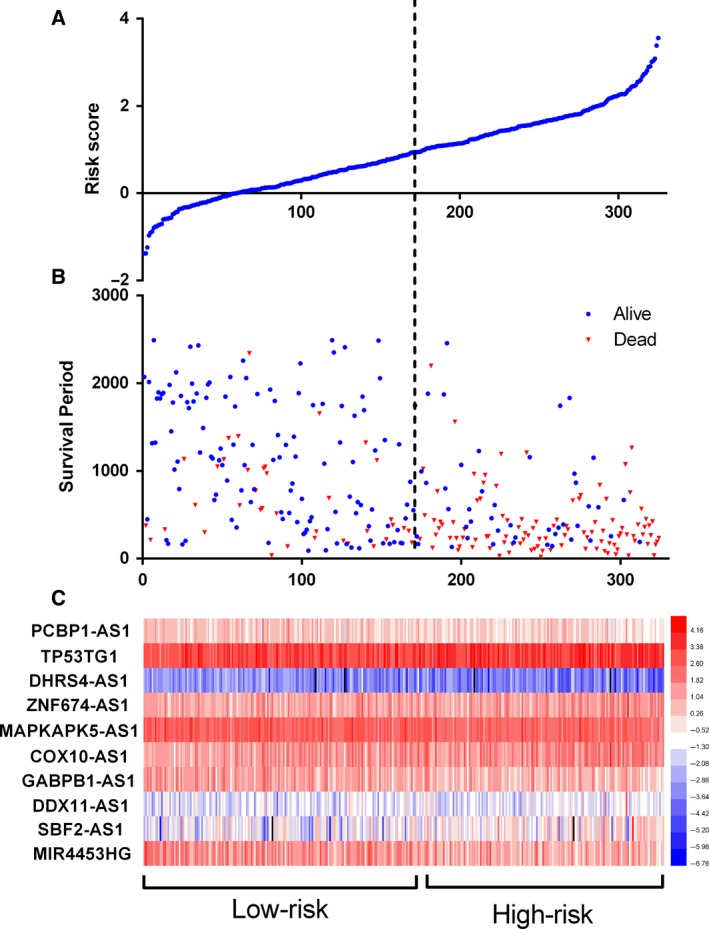
Autophagy‐related lncRNA risk score analysis of glioma patients in CCGA. (A) The low and high score group for the autophagy‐related lncRNA signature in glioma patients. (B) The survival status and duration of glioma cases. (C) Heatmap of the 10 key lncRNAs expression in glioma. The color from blue to red shows an increasing trend from low levels to high levels.

**Figure 4:**
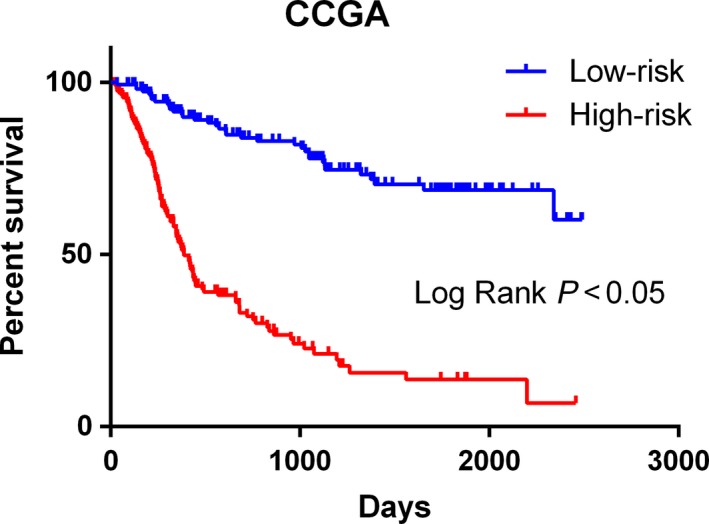
Kaplan–Meier survival curves for the autophagy‐related lncRNA risk score for glioma in CCGA dataset. The Kaplan–Meier survival curves showed that the OS period is longer in the low‐risk group than that in the high‐risk group in the CCGA datasets (median OS 1211 *vs* 346 days; log rank *P* < 0.05).

Next, we use a risk score method to develop an autophagy‐related lncRNA signature. We divided the glioma patients into two groups (low‐risk group and high‐risk group) by median risk score (Fig. 3). As a result, the risk score could significantly predict the OS of glioma patients, in which the OS period is longer in the low‐risk group than that in the high‐risk group (median OS 1211 *vs* 346 days; log rank *P* < 0.05). Additionally, the Cox regression analysis also revealed a significant prognostic effect of the risk score on the glioma patients (HR = 5.307, 95% CI: 4.195–8.305; *P* < 0.0001, Fig. 4). Further, we also explored whether the risk score signature is an independent predictor for the prognosis of glioma patients by multivariate Cox regression analysis. As a consequence, a HR of 2.736 indicated that the risk score could significantly contribute to the prediction of survival of glioma patients, eliminating the influence of other factors such as sex, age, grade, radiotherapy, chemotherapy and the molecular status (*IDHDampR*,* TP53.1*,* EGFR*,* ATRX* and *EZH2*) (Table 3).

**Table 3:** Multivariate Cox regression analysis of characteristics and risk score in glioma

Variable	β	SE	Wald	*P*	HR	Lower	Upper
Gender	−0.022	0.221	0.01	0.921	0.978	0.635	1.507
Age	0	0.01	0	0.987	1	0.98	1.021
Grade	0.831	0.16	26.965	< 0.001	2.296	1.678	3.143
Radiotherapy	−0.932	0.206	20.553	< 0.001	0.394	0.263	0.589
Chemotherapy	−0.469	0.207	5.143	0.023	0.626	0.417	0.938
*IDHDampR*	−0.639	0.248	6.641	0.01	0.528	0.324	0.858
*TP53.1*	−0.334	0.186	3.23	0.072	0.716	0.498	1.031
*EGFR*	−0.165	0.212	0.605	0.437	0.848	0.559	1.285
*ATRX*	−0.817	0.41	3.98	0.046	0.442	0.198	0.986
*EZH2*	0.477	0.271	3.104	0.078	1.612	0.948	2.742
Risk score	1.006	0.243	17.156	< 0.001	2.736	1.699	4.405

### Clinical value of the lncRNA signature for glioma patients

Subsequently, we also determined the clinical value of the 10‐lncRNA signature regarding the grade, radiotherapy and chemotherapy. As shown in the Table 4, the risk score tends to increase in the higher grades, suggesting that this lncRNA signature might be associated with the progression of glioma. Interestingly, the risk score was lower in patients receiving radiotherapy than that in patients without radiotherapy (*t* = −2.267, *P* = 0.025). In contrast to the results of radiotherapy, a higher risk score was found in patients without chemotherapy, while the patients who had received chemotherapy presented a lower risk score. Moreover, we also assessed differences in risk score based on molecular status. As a result, lower risk scores were found in those with the *IDH* mutation than in those without, indicating a potential association between the lncRNA signature and *IDH* mutation.

**Table 4:** Clinical impact of risk score signature for the CCGA cohort

Clinicopathological feature	*n*	Risk score			
		Mean	SD	*t*	*P*
Grade					
I–II	216	1.203847424	0.8808	10.898	< 0.001
III–IV	109	0.245607385	0.6717		
Radiotherapy					
Yes	212	0.777059137	0.9674	−2.267	0.025
No	84	1.029456602	0.8189		
Chemotherapy					
Yes	158	1.01284085	0.8665	2.604	0.01
No	128	0.727299406	0.9862		
IDH (DNA and RNA)					
Mutation	171	0.497626264	0.8174	−8.69	< 0.001
Wildtype	154	1.30979323	0.8671		
IDH1‐R32					
Wildtype	162	0.509472199	0.8242	−7.824	< 0.001
Mutation	163	1.253176395	0.8881		
*TP53.1*					
Wildtype	189	0.937494898	0.9505	1.254	0.211
Mutation	136	0.805997888	0.9062		
*EGFR*					
Wildtype	110	0.770831973	0.9802	−1.546	0.123
Mutation	215	0.939584798	0.905		
*ATRX*					
Wildtype	33	0.856142312	0.9358	−0.171	0.865
Mutation	292	0.885443672	0.9343		
*EZH2*					
Wildtype	37	1.182088779	1.1162	1.77	0.084
Mutation	288	0.843975569	0.9019		

### Validation in the TCGA dataset

**Figure 5:**
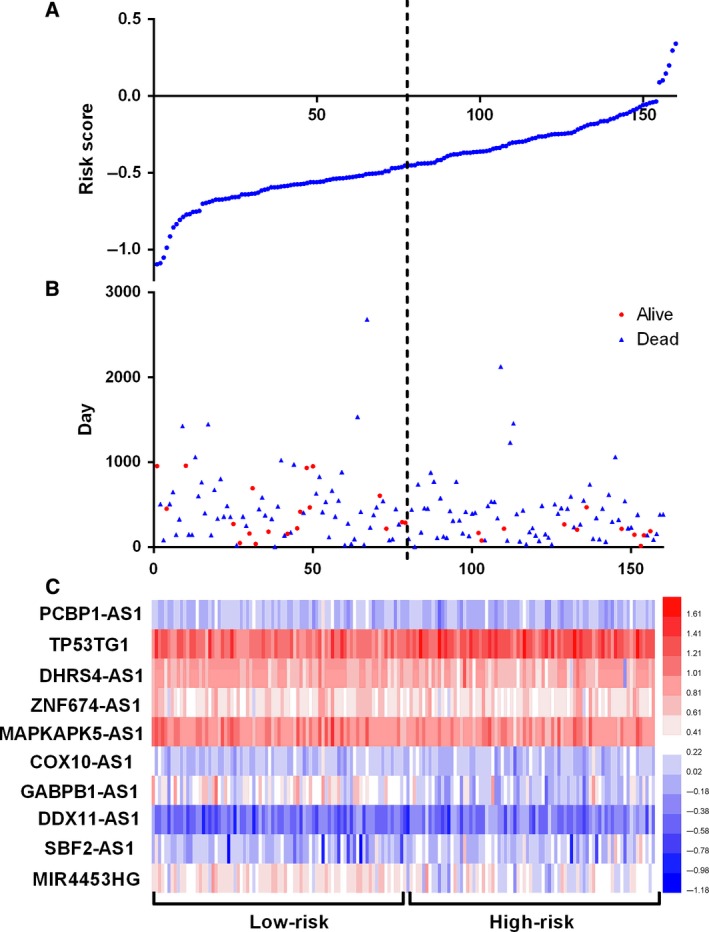
Autophagy‐related lncRNA risk score analysis of glioma patients in TCGA. (A) The low and high score group for the autophagy‐related lncRNA signature in glioma patients. (B) The survival status and duration of glioma cases. (C) Heatmap of the 10 key lncRNAs expressed in glioma. The color from blue to red shows an increasing trend from low levels to high levels.

**Figure 6:**
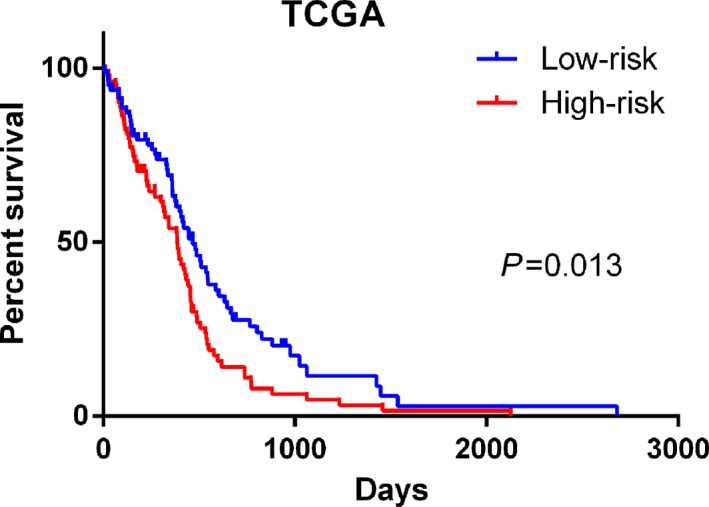
Kaplan–Meier survival curves for the autophagy‐related lncRNA risk score for glioma in TCGA dataset. Consistent with the results derived from the CGGA dataset, the high‐risk patients had a shorter median OS than that of the low‐risk patients in TCGA datasets (median OS 385 *vs* 468 days; log rank *P* = 0.012).

Next, these results were further validated in the additional dataset (TCGA) using the same β value. In total, 160 GBM patients were enrolled for the validation of the lncRNA signature (Fig. 5). We divided these patients into the high‐risk and low‐risk groups on the basis of the median value of the risk score. Consistent with the results derived from the CGGA dataset, the high‐risk patients had a shorter median OS than that of the low‐risk patients (median OS 385 *vs* 468 days; log rank *P* = 0.012; Fig. 6). This finding was further validated by Cox regression analysis, in which the high‐risk group tended to have a shorter OS time for GBM patients than that of the low‐risk group (HR = 1.544, 95% CI: 1.110–2.231; *P* = 0.031). In light of these results, we could confirm that the lncRNA signature provides a robust prediction for the prognosis of glioma patients.

### Gene set enrichment analysis

**Figure 7:**
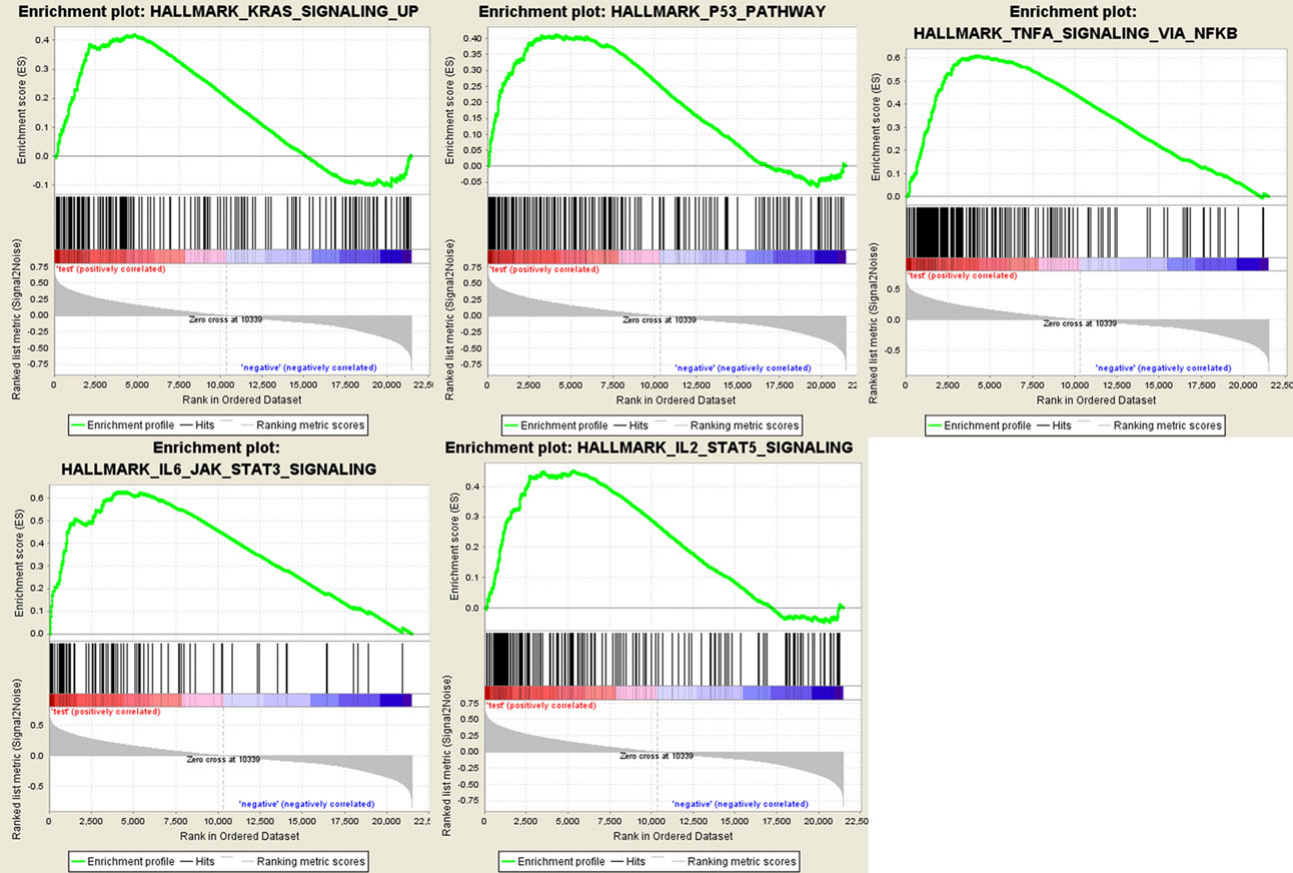
Gene set enrichment analysis indicated significant enrichment of hallmark cancer‐related pathways in the high‐risk group based on CCGA dataset. JAK, Janus kinase; NFKB, nuclear factor‐κB; TNFA, tumour necrosis factor α.

**Figure 8:**
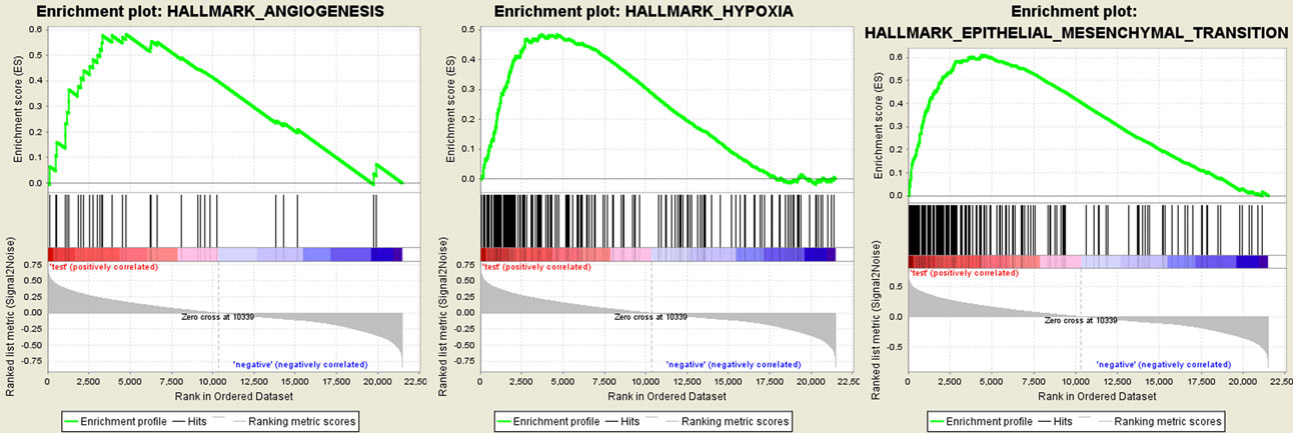
Gene set enrichment analysis indicated significant enrichment of the progression‐ and metastasis‐related pathway in the high‐risk group based on CCGA dataset.

**Figure 9:**
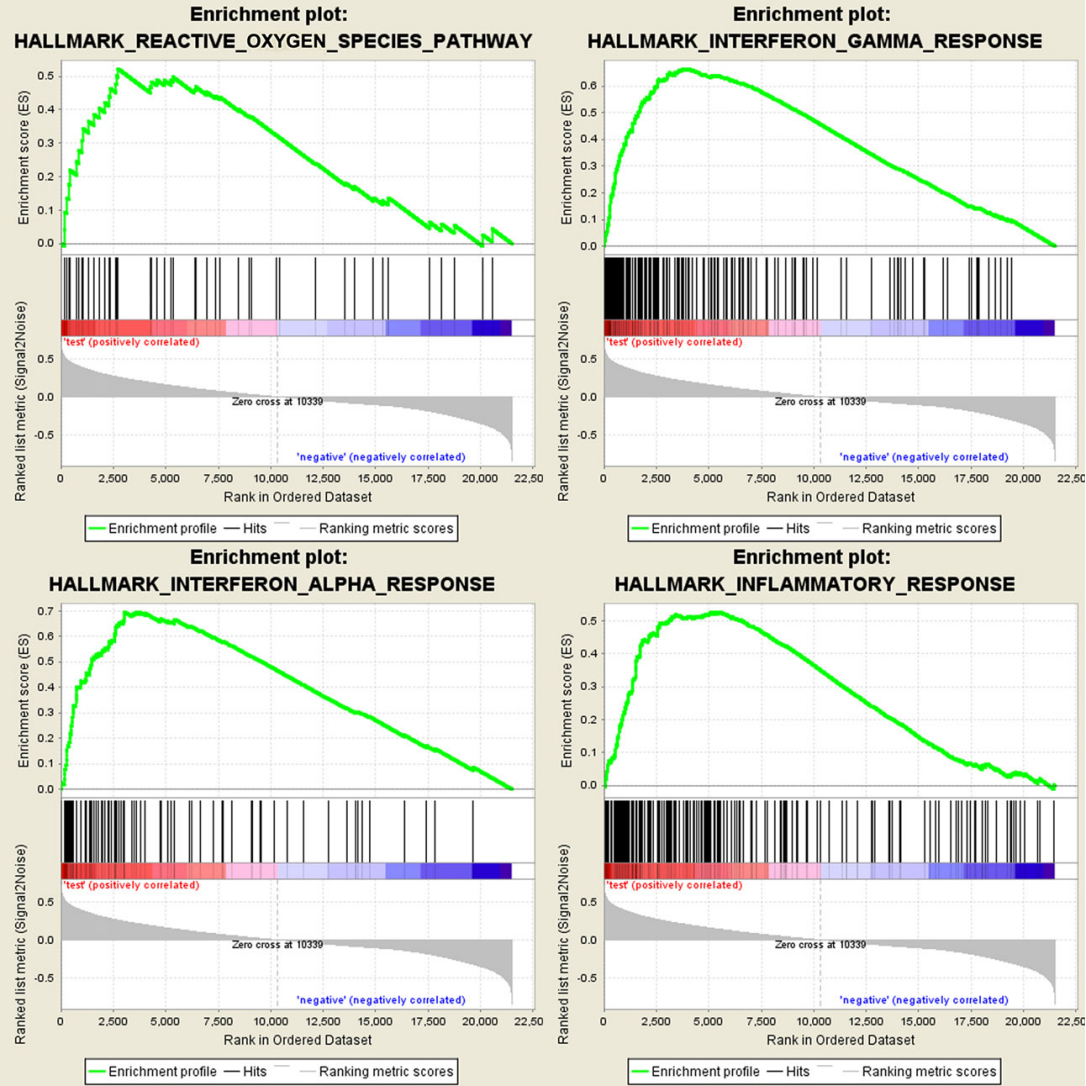
Gene set enrichment analysis indicated significant autophagy‐related enrichment based on CCGA dataset.

Further functional annotation was conducted through GSEA. The results revealed that the differentially expressed genes between the two groups were enriched in the autophagy‐related and tumor‐related pathways. As result, a total of 19 gene sets were significantly enriched at a nominal *P*‐value < 5% (Table 5). Among the gene sets, several pathways are well‐established in cancers, including interleukin (IL) 6/Janus kinase/signal transducer and activator of transcription (STAT) 3 signaling, tumor necrosis factor α signaling via nuclear factor‐κB, IL2/STAT5 signaling, the p53 pathway and the KRAS signaling pathway (Fig. 7). Moreover, the gene sets were also found to be involved in the vital functions of tumorigenesis and progression of cancer. For instance, epithelial mesenchymal transition, angiogenesis and hypoxia were closely related to the invasion and metastasis of cancer (Fig. 8). Notably, the GSEA revealed that the gene sets were involved in the reactive oxygen species pathway, interferon (IFN)‐γ response, IFN‐α response and inflammatory response, which are strongly associated with autophagy (Fig. 9). Taken together, the defined autophagy‐related genes contribute to vital cancer and autophagy pathways, which might provide strong evidence for a cancer‐targeted treatment for glioma.

**Table 5:** Gene set enrichment analysis results based on the signature of 10 autophagy lncRNAs

Name	Size	ES	NES	NOM *P*‐value	FDR *q*‐value	FWER *P*‐value	Rank at max	Leading edge
Hallmark_Interferon_gamma_response	194	0.663942	2.002969	0.003831	0.03201	0.019	3784	tags = 62%, list = 18%, signal = 74%
Hallmark_Coagulation	134	0.546977	1.968419	0	0.024199	0.027	4273	tags = 47%, list = 20%, signal = 58%
Hallmark_Allograft_rejection	196	0.606039	1.934347	0.005894	0.02353	0.037	4277	tags = 59%, list = 20%, signal = 73%
Hallmark_Epithelial_mesenchymal_transition	195	0.61046	1.914759	0.01354	0.02293	0.051	4360	tags = 62%, list = 20%, signal = 77%
Hallmark_Interferon_alpha_response	95	0.695722	1.901815	0.007937	0.020948	0.057	3003	tags = 61%, list = 14%, signal = 71%
Hallmark_Il6_jak_stat3_signaling	86	0.627249	1.854703	0.011905	0.02949	0.083	4232	tags = 60%, list = 20%, signal = 75%
Hallmark_Tnfa_signaling_via_nfkb	197	0.609462	1.79719	0.024	0.041906	0.129	4243	tags = 58%, list = 20%, signal = 72%
Hallmark_Angiogenesis	35	0.582243	1.740052	0.005988	0.059463	0.178	4728	tags = 57%, list = 22%, signal = 73%
Hallmark_Complement	192	0.472855	1.732826	0.026263	0.056876	0.188	3996	tags = 45%, list = 19%, signal = 55%
Hallmark_Hypoxia	197	0.484747	1.725543	0.034068	0.053391	0.195	3666	tags = 45%, list = 17%, signal = 54%
Hallmark_Glycolysis	194	0.445341	1.707246	0.014	0.055343	0.223	4979	tags = 49%, list = 23%, signal = 64%
Hallmark_Il2_stat5_signaling	196	0.451793	1.706509	0.017341	0.050981	0.223	5329	tags = 54%, list = 25%, signal = 71%
Hallmark_Reactive_oxigen_species_pathway	46	0.522081	1.675037	0.017964	0.061012	0.263	2719	tags = 39%, list = 13%, signal = 45%
Hallmark_Inflammatory_response	194	0.527449	1.670621	0.037549	0.057958	0.266	5277	tags = 59%, list = 25%, signal = 77%
Hallmark_P53_pathway	195	0.411785	1.645334	0.027559	0.063102	0.302	4075	tags = 38%, list = 19%, signal = 47%
Hallmark_Kras_signaling_up	198	0.419712	1.634183	0.028	0.063116	0.318	4808	tags = 51%, list = 22%, signal = 64%
Hallmark_Apoptosis	159	0.432405	1.616621	0.023529	0.066577	0.339	5324	tags = 53%, list = 25%, signal = 71%
Hallmark_Apical_surface	44	0.402856	1.481723	0.034483	0.132731	0.539	1498	tags = 23%, list = 7%, signal = 24%
Hallmark_Mtorc1_signaling	193	0.417297	1.473019	0.094378	0.130749	0.55	4257	tags = 44%, list = 20%, signal = 54%
Hallmark_Apical_junction	195	0.35423	1.457525	0.066148	0.133971	0.567	3822	tags = 33%, list = 18%, signal = 40%

ES, enrichment score; FDR, false discovery rate; FWER, familywise‐error rate; NES, normalized enrichment score; NOM *P* Value, nominal *P* Value.

## Discussion

Glioma is the most aggressive and common type of primary brain tumor in humans. With the development of clinical management of glioma, some prognostic factors are well characterized, including tumor size, tumor grade and stage. High‐throughput biological technologies are being widely used to predict cancer recurrence and tumor metastasis by detecting the alteration of miRNAs or genes 23, 24. The major class of lncRNAs, as a complement to genes or miRNAs, provides a promising opportunity to predict the risk of recurrence of glioma 25. However, so far, there has been no systematic process to identify lncRNA signature sets for predicting the survival of glioma patients. Therefore, it is necessary to establish a lncRNA signature to predict the prognosis of glioma patients.

In this study, two datasets (CGGA and TCGA) were collected to explore the prognosis of autophagy‐related lncRNAs for glioma patients. In the first step, we identified 402 lncRNAs through the lncRNA–autophagy gene co‐expression network. Furthermore, we identified 10 autophagy‐associated lncRNA signatures that could divide glioma patients into high‐ and low‐risk groups based on the median risk score. Additionally, it was found that the OS is longer in the low‐risk group than that in the high‐risk group. Through univariate and multivariate Cox regression analyses, we can conclude that the signature is an independent factor that is significantly related to OS.

Although little is known about the role of autophagy in cancer therapy to date, recent studies suggest that autophagy therapy will become a new approach to glioma treatment 26, 27. In recent studies, IFN‐γ was found to influence autophagy and cell growth in human hepatocellular carcinoma (HCC) cells. IFN‐γ is a cytokine with anti‐viral and immune regulation. The cytokine induces autophagosome formation and transformation of microtubule‐associated protein 1 light chain 3 proteins and can inhibit cell growth and non‐apoptotic cell death in Huh7 cells. In addition, autophagy in Huh7 cells is also activated by the overexpression of interferon‐regulatory factor‐1. Eventually, induced autophagy will inhibit IFN‐γ and cell death in HCC 28. Since autophagy can respond to a variety of stresses to promote the survival of cancer cells, it has protumorigenic functions. Glucose metabolism promotes adhesion‐independent conversion driven by oncogene insult‐mutationally active Ras. In human cancer cell lines carrying KRAS mutations and cells ectopically expressing oncogenic H‐Ras, autophagy is induced after the extracellular matrix is isolated. If autophagy is inhibited by RNA interference‐mediated depletion of multiple autophagic regulators or genetic deletion, Ras‐mediated conversion and glycolytic capacity proliferation independent of adhesion will be impaired. In addition, when the availability of glucose is decreased, the conversion and proliferation of autophagy‐deficient cells expressing oncogenic Ras are unaffected, which is just the opposite of that in autophagy‐competent cells. In conclusion, autophagy can promote the unique mechanism of Ras‐driven tumor growth in specific metabolic environments 29.

Among the 10 autophagy‐related lncRNAs, *PCBP1‐AS1*,* DHRS4‐AS1*,* MAPKAPK5‐AS1* and *GABPB1‐AS1* were risk‐associated genes, while *TP53TG1*,* ZNF674‐AS1*,* DDX11‐AS1*,* SBF2‐AS1*,* MIR4453HG* and *COX10‐AS1* were protective genes. Specifically, we also found that the high‐risk group was enriched in the glycolysis pathway. Consistent with our studies, a recent study revealed that *TP53TG1* might affect the expression of glucose metabolism‐related genes under glucose deprivation, leading to cell proliferation and migration of glioma cells 30. Additionally, *MAPKAPK5‐AS1* regulates gene expression by acting with miRNAs and is significantly associated with the OS of liver cancer 31. Furthermore, the expression of *COX10‐AS1* in oral cavity and oropharyngeal squamous cell carcinoma is more than twice that of normal cells 32.

All of the lncRNAs we identified directly or indirectly regulate autophagy, many by regulating miRNAs; thus, we must perform lncRNA–mRNA co‐expression analyses to assess the function of lncRNAs 33, 34, 35. Therefore, we can conclude that due to the various functions of lncRNAs, the 10 autophagy‐related lncRNAs we identified will be potential therapeutic targets 12, 36.

In conclusion, by constructing an autophagy–lncRNA coexpression network, we identified a signature of 10 autophagy‐related lncRNAs, which has prognostic value for glioma patients. In addition, our study classified low‐risk and high‐risk groups based on the median risk score, and each showed different autophagy states.

## Conflict of interest

The authors declare no conflict of interest.

## Author contributions

LM designed the study, and revised the manuscript. FL, the main author of study, conceived and designed the analysis and wrote the manuscript. WC and MC took part in analyzing the data and writing the manuscript. JY and HC analyzed the data and conducted the results. HY and TL contributed to writing and revising the manuscript. All authors read and approved the final manuscript.
